# Layered Double Hydroxide as a Potent Non-viral Vector for Nucleic Acid Delivery Using Gene-Activated Scaffolds for Tissue Regeneration Applications

**DOI:** 10.3390/pharmaceutics12121219

**Published:** 2020-12-16

**Authors:** Lara S. Costard, Domhnall C. Kelly, Rachael N. Power, Christopher Hobbs, Sonia Jaskaniec, Valeria Nicolosi, Brenton L. Cavanagh, Caroline M. Curtin, Fergal J. O’Brien

**Affiliations:** 1 Tissue Engineering Research Group, Department of Anatomy and Regenerative Medicine, Royal College of Surgeons in Ireland (RCSI), 123 St Stephen’s Green, D02 YN77 Dublin, Ireland; laracostard@rcsi.com (L.S.C.); domhnallkelly@rcsi.ie (D.C.K.); rachaelnpower@rcsi.ie (R.N.P.); 2 Centre for Research in Medical Devices (CÚRAM), Biomedical Sciences Building, National University of Ireland, Galway (NUI, Galway), H91 TK33 Galway, Ireland; 3 Advanced Materials and Bioengineering Research Centre (AMBER), RCSI and Trinity College Dublin (TCD), College Green, D02 PN40 Dublin, Ireland; hobbsc@tcd.ie (C.H.); metels@tcd.ie (S.J.); NICOLOV@tcd.ie (V.N.); 4 School of Chemistry and Centre for Research on Adaptive Nanostructures and Nanodevices (CRANN), Trinity College Dublin, College Green, D02 PN40 Dublin, Ireland; 5 Cellular and Molecular Imaging Core, RCSI, 123 St Stephen’s Green, D02 YN77 Dublin, Ireland; brentoncavanagh@rcsi.ie; 6 Trinity Centre for BioMedical Engineering, Trinity Biomedical Sciences Institute, TCD, College Green, D02 PN40 Dublin, Ireland

**Keywords:** biomaterials, scaffold, nonviral vector, gene therapy, layered double hydroxide, microRNA, siRNA, plasmid DNA, drug delivery

## Abstract

Nonviral vectors offer a safe alternative to viral vectors for gene therapy applications, albeit typically exhibiting lower transfection efficiencies. As a result, there remains a significant need for the development of a nonviral delivery system with low cytotoxicity and high transfection efficacy as a tool for safe and transient gene delivery. This study assesses MgAl-NO_3_ layered double hydroxide (LDH) as a nonviral vector to deliver nucleic acids (pDNA, miRNA and siRNA) to mesenchymal stromal cells (MSCs) in 2D culture and using a 3D tissue engineering scaffold approach. Nanoparticles were formulated by complexing LDH with pDNA, microRNA (miRNA) mimics and inhibitors, and siRNA at varying mass ratios of LDH:nucleic acid. In 2D monolayer, pDNA delivery demonstrated significant cytotoxicity issues, and low cellular transfection was deemed to be a result of the poor physicochemical properties of the LDH–pDNA nanoparticles. However, the lower mass ratios required to successfully complex with miRNA and siRNA cargo allowed for efficient delivery to MSCs. Furthermore, incorporation of LDH–miRNA nanoparticles into collagen-nanohydroxyapatite scaffolds resulted in successful overexpression of miRNA in MSCs, demonstrating the development of an efficacious miRNA delivery platform for gene therapy applications in regenerative medicine.

## 1. Introduction

Recent developments in the understanding of the mechanism of action of gene therapies, together with improved safety measures, has led to the expansion of their use for treatments for rare, life-threatening genetic disorders, to the potential widespread use in the treatment of more common diseases and disorders [1]. Furthermore, gene therapy has been used as an alternative approach to protein delivery in regenerative medicine applications, including the promotion of both bone and cartilage repair [2,3], while offering a potential method of overcoming the limitations and adverse events associated with recombinant protein delivery. Collagen-based biomaterial scaffolds optimized in our group for tissue repair have also shown the potential to locally deliver genes and thereby provide a promising tool to limit the adverse effects of gene therapy [4,5,6,7,8,9,10].

Conventionally, gene therapy uses the delivery of plasmid DNA (pDNA) to express a gene of therapeutic benefit [11]. This approach uses the cell itself as the factory to produce a particular protein(s) of interest. Additionally, gene therapy can also enable the silencing of specific proteins, which under normal circumstances may negatively affect the tissue regeneration process through the delivery of small interfering (si)RNA or micro (mi)RNA to harness the therapeutic potential of the RNA interference (RNAi) pathway. Specifically, miRNAs are small, single-stranded RNA, which can bind to multiple messenger RNA (mRNA) targets with imperfect complementarity and inhibit their translation [12]. They are involved in several physiological and pathological processes and therefore serve as interesting therapeutic targets in multiple areas, including bone repair [4,13], cancer [14] and cardiac diseases [15]. Native miRNAs can be overexpressed by the delivery of miRNA mimics carrying the same sequence as the endogenous miRNA, or can be suppressed using hairpin inhibitors carrying a complementary sequence of the endogenous miRNA, and therefore binding and inhibiting the miRNA’s action [4,16]. Similarly, siRNA binds to specific mRNA sequences with strict complementarity and inhibits its expression [17]. The clinical potential of targeting the RNAi pathways is highlighted by the recent FDA approval of ONPATTRO/Patisiran, which uses a lipid-based siRNA nanoparticle complex for the treatment of hereditary transthyretin (hATTR) amyloidosis [18].

While gene therapy offers huge potential as disease treatment, there are several barriers in the translation of safe and effective delivery of nucleic acid cargo to target cell populations, which have impeded its widespread clinical use [19]. Unfavorable physicochemical properties and poor biodistribution profiles of nucleic acids (pDNA, mRNA, siRNA or miRNA) significantly hinder the ease and effectiveness of the delivery of these molecules to target cell populations [11]. To facilitate optimum delivery and functionality of these molecules, delivery vectors, which can be either viral or nonviral, are required. To date, viral vectors have demonstrated greater clinical success, largely due to higher transfection efficiency [20]. Despite this, safety concerns regarding the use of such vectors remain, including potential issues regarding immunogenicity and integration of genetic cargo into the genome resulting in undesirable sustained/over-production of proteins [21]. Nonviral gene delivery, although often demonstrating lower levels of transfection efficiency, can be used to overcome many of these issues while also offering several notable advantages over viral counterparts, including ease of large-scale manufacturing and cost-effectiveness [22,23,24]. To date, several nonviral vectors have demonstrated successful delivery of nucleic acid cargoes to cells safely and effectively, including lipid-based vectors [25,26], cationic polymers [27], cationic polysaccharides [28], cell-penetrating peptides [28], calcium phosphates [2] and cyclodextrins [29]. Despite this success, there is yet to be a consensus on an “optimal” nonviral vector for the delivery of nucleic acid cargo. It is likely that different vectors will be required depending on several factors, including the target cell type and the overall application for which the treatment is being applied [19,30]. Of particular interest is the effect that the different physicochemical characteristics of each cargo (pDNA, siRNA, miRNA), as well as the site of action for each cargo, has on determining the choice of the delivery vector with regards to both safety and efficiency. With this in mind, the delivery of pDNA is especially challenging, as pDNA needs to translocate into the nucleus to enable transcription of the encoded protein, whereas both siRNA and miRNA act within the cytoplasm [31]. Furthermore, the significantly larger size and accompanying negative charge associated with pDNA compared to that of smaller siRNA and miRNA molecules presents a further challenge where the physical structure and condensing properties of a vector necessary for pDNA delivery may not be suitable for the delivery of siRNA and miRNA, and vice versa [32].

Innovative approaches in drug delivery have been fueled by new findings within the field of nanotechnology. One such example is the emergence of layered double hydroxides (LDHs) as nonviral delivery vectors. LDHs are two-dimensional materials of cationic layers incorporating anions. Positively charged layers are made of magnesium and aluminum hydroxides, while the interlayer space is filled with negative species (such as nitrate or carbonate) [33], and have previously been utilized as nonviral vectors to deliver anticancer drugs, such as methotrexate or 5-fluorouracil [34,35,36,37,38] and antisense oligonucleotides [39]. Broadly speaking, ion exchange of the interlayer ions allows LDH to interact with negatively charged biomolecules such as anionic nucleic acids. An overall positive surface charge of LDH–nucleic acid complexes enables further interaction and attraction to the anionic plasma membrane of the cell. To date, successful formulation and delivery of Mg2Al LDH–siRNA nanoparticles have been demonstrated with the transfection of human embryonic kidney (HEK 293T) cells [40], as well as cortical neurons [32]. Mg/Ga LDH has been utilized to deliver pDNA as well as DNA fragments to HeLa cells [41]. However, delivery of pDNA, as well as supercoiled DNA, has proven difficult, with a requirement for high amounts of LDH needed to encapsulate the DNA. This requirement for increased amounts of LDH, compared to that used to complex with smaller nucleic acid molecules, can result in cytotoxic effects [42]. Cytotoxic effects of Mg2Al-based LDH have been reported for doses over 200 mg/L for the delivery of supercoiled pDNA to HEK 293T cells [42]. However, these effects appear to vary based on cell type. For example, HL-60 human leukemia cells demonstrated a higher tolerance for Mg2Al-LDH [39]. Interestingly, size-dependent cytotoxicity has also been reported with small LDH nanoparticles (~50 nm), demonstrating a higher degree of cytotoxicity compared to larger LDH nanoparticles (100–200 nm) [43]. The variation in both the efficacy and cytotoxicity of LDH nanoparticles as a result of the specific nucleic acid cargo used suggests that LDH nanoparticles may have a niche functionality allowing for efficient miRNA and siRNA delivery, providing a safe delivery pathway while minimizing the need for nuclear translocation.

Another important consideration in the delivery of gene therapies is the spatiotemporal aspect of delivering nucleic acid molecules. It is important to note that even with a suitable delivery vector, complexes often undergo rapid clearance from the site of action. Methods of local delivery of nucleic acid cargo/complexes provide numerous advantages over systemic administration, in addition to increased efficiency. The use of biomaterial-based scaffolds is a successful method of providing a platform for advanced delivery of nucleic acid complexes while also offering an extra degree of protection from serum nucleases and rapid clearance, enabling localized delivery of nucleic acids to a specific environment. Additionally, the therapeutic effect of nucleic acid delivery has been shown to be time-dependent, which highlights the importance of using a platform, which allows for the tailored release of the therapeutic in a sustained, yet ultimately transient manner [4]. Furthermore, the characteristics and composition of these biomaterial-based scaffolds can be modified to provide desirable structural support and mechanical properties tailored to a specific tissue type, providing a further enhancement of the endogenous mechanisms of repair and regeneration [4,44]. Previous research within our group has highlighted the potential of such platforms for use in various tissue engineering and cancer applications using a variety of nonviral vectors and scaffold compositions [2,3,4,45].

This study investigates the suitability of a MgAl-NO3 layered double hydroxide as a nonviral delivery vector for pDNA, siRNA, miRNA and assesses the potential of LDH–nucleic acid loaded collagen-based scaffolds as advanced delivery systems for safe, efficient nucleic acid delivery to MSCs. Collagen-nanohydroxyapatite (coll–nHA) scaffolds have been used in our group to promote bone repair and to locally deliver nucleic acids for tissue regeneration applications [2,4,5]. While LDHs have been previously used as components of biomaterials for bone regeneration applications in combination with hydroxyapatite for the release of Vitamin D3 from hydroxyapatite/gelatine scaffolds [46,47], as gene delivery vectors, they have primarily been used to transfect cancer cells [48,49,50,51], other cell lines such as HEK 293T [52], NIH-3T3 and HeLa cells [53] as well as neurons [54,55], but also for siRNA delivery to chondrocytes [56]. In our study, we describe, for the first time, the use of LDH for the delivery of miRNAs to MSCs, indicating their potential as gene delivery vectors in bone repair applications. We first determined the ability of the cationic LDH structures to complex with various nucleic acid molecules of different sizes and physicochemical properties, including a 4200 bp plasmid DNA and smaller (~22 bp) siRNA and miRNA sequences. Following the successful formulation of LDH–nucleic acid complexes, in vitro transfection efficiency screening was carried out to assess cytocompatibility, cellular uptake, and functionality of LDH–nucleic acid delivery to MSCs. Finally, optimized formulations of LDH–nucleic acid complexes were used for the development of 3D collagen-LDH–loaded scaffolds to test the efficiency of these advanced 3D delivery systems.

## 2. Materials and Methods

### 2.1. Synthesis of MgAl-NO_3_ LDH Nanoparticles

Aluminum nitrate nonahydrate (≥98%), sodium hydroxide (≥97%) and sodium nitrate (≥99%) were obtained from Sigma-Aldrich. Magnesium nitrate hexahydrate (99%) was purchased from Merck. All chemicals were used as received without further purification.

Mg-Al LDH delivery vectors were prepared by the co-precipitation method. Briefly, the precursor’s solution was prepared by dissolving 3.75 mol of Mg(NO_3_)2·6H_2_O and 1.25 mol Al(NO_3_)·9H_2_O in 100 mL deionized water (total metals concentration equal to 0.5 M). Then, the solution was drop added to an equal volume of 1.1 M NaOH containing 0.15 M NaNO_3_ under vigorous stirring and N2 bubbling. After precipitation, the suspensions were centrifuged at 3000 rpm for 20 min, redispersed in water, diluted 1:10 and aged at room temperature for 7 days.

### 2.2. Complexation of LDH with Nucleic Acid (pDNA, siRNA, miRNA)

MgAl-NO_3_ (1.76 mg/mL) was sonicated for 1 h before use. LDH–nucleic acid particles were formulated at a series of mass ratios (MR) of LDH:nucleic acid, as indicated in the following subsections. Briefly, known amounts of nucleic acid, added to either molecular grade H_2_O (for physicochemical analysis) or OptiMEM (for cell studies), were added to LDH at the desired MR. Particles complexed via electrostatic interactions between negatively charged nucleic acids and positively charged LDH were incubated for 15 min at room temperature prior to experimentation.

#### 2.2.1. Plasmid DNA (pDNA) Propagation

Plasmid encoding Gaussia luciferase (pLuc; New England Biolabs, Massachusetts, USA) under the control of the cytomegalovirus promoter was propagated by transforming IBA solutions for Life Sciences One Shot™TOP10 chemically competent *E. coli* (Fisher Scientific Ireland, Dublin, Ireland) according to the manufacturer’s protocol. Transformed cells were then expanded on lysogeny broth (LB) plates containing 100 µg/mL ampicillin (Sigma-Aldrich, Arklow, Ireland). After a 24h incubation at 37 °C, the resultant colonies were harvested and amplified in LB broth containing 100 µg/mL ampicillin overnight in a shaking incubator at 37 °C and 225 rpm. DNA was purified and collected using the Endotoxin-free Maxi-prep kit (Qiagen, Manchester, UK). The plasmid was dissolved in molecular grade H_2_O (Sigma-Aldrich, Arklow, Ireland) at a concentration of 1 μg/mL and stored at −20 °C.

#### 2.2.2. Physicochemical Nanoparticle Characterization

Particle size and polydispersity index were characterized by dynamic light scattering (DLS), while electrophoretic light scattering (ELS) was used to measure the zeta potential of LDH–nucleic acid complexes (pDNA, siRNA, miRNA) (Zetasizer 3000 HS, Malvern Instruments, UK). LDH–nucleic acid complexes were prepared as outlined above. After complexation, the solution volume was brought to 1 mL using molecular grade H_2_O and transferred to a zeta-cell (Malvern, UK) for analysis. Measurements were performed at 25 °C with a 100 mW laser set at 90° to the sample.

#### 2.2.3. Nucleic Acid Encapsulation

Encapsulation efficiency refers to the ability of a vector to encapsulate nucleic acid cargo, thereby forming a nanoparticle that protects the nucleic acid from nucleases in the surrounding extracellular environment.

pDNA encapsulation efficiency was measured by complexing pLuc pDNA with the LDH, prior to staining pDNA with the QuantiFluor^®^ dsDNA System (Promega Corporation, Southhampton, UK) as per the manufacturer’s protocol, before complexation with LDH at MR10, 50, 75 and 100, as described in Section 2.2. A solution containing pDNA alone was complexed with the stain as a positive control, while a solution containing 1X TE buffer and QuantiFluor^®^ stain alone was the negative control for this assay. Following complexation, 100 µl of each control and sample were pipetted into a Corning Costar^®^96-well black polystyrene plate, and fluorescence was measured on a Tecan Infinite^®^200 Pro plate reader at excitation wavelength 504 nm and emission wavelength 531 nm. The assay was carried out after initial complexation and 5 h later. Encapsulation efficiency was calculated by normalizing the fluorescence in the uncomplexed pDNA sample to 100% exposure and subtracting the percentage exposure for each sample from 100%.

Encapsulation efficiency and stability of RNA-nanoparticles (siRNA and miRNA) were assessed using the Quant-iT RiboGreen (BioSciences, Dublin, Ireland) according to the manufacturer’s protocol. Fluorescence was read at 520 nm using a VarioskanFlash plate reader (Thermo Scientific, Germany) and SkanIt RE for VarioSkan software. Free RNA content after complexation was compared to RNA content after heparin digestion of nanoparticles to calculate the percentage of encapsulated RNA. Stability at 37 °C was assessed by measuring encapsulated RNA immediately and 5 h after complexation. Nanoparticles with the optimal physicochemical properties were taken forward for use in further in vitro characterization.

### 2.3. Mesenchymal Stromal Cell Uptake and Cytocompatibility of LDH–Nucleic Acid Complexes in 2D Monolayer

Rat mesenchymal stromal cells (MSCs) were isolated from the bone marrow of 8-week-old male Wistar rats (approved under TH017, 27.05.2019) and cultured in fully supplemented rat MSC medium (Dulbecco’s modified Eagle’s medium (DMEM) containing 20% fetal bovine serum (FBS), 1% non-essential amino acids, 1% GlutaMAX, and 0.2% Primocin. Rat MSCs were characterized as described previously [57]. Cells were seeded at a density of 5208 cells/cm^2^ 24 h before transfection. One hour before transfection, the growth medium was exchanged for a serum-free medium (OptiMEM). LDH–nucleic acid complexes were formulated as described in Section 2.1 and added to the cells. Mass ratio, incubation time and nucleic acid dose were varied to determine optimal transfection conditions. After the desired incubation period, transfection media was removed, cells were washed twice in phosphate-buffered saline (PBS), and fresh growth media was added to the cells. Growth media was replenished every 3 to 4 days for the duration of the experiments.

#### 2.3.1. Cellular Uptake

pDNA: reporter plasmid pLuc (New England Biolabs, Massachusetts, USA) was tagged with Cy3 nucleic acid tag, as per the manufacturer’s instructions. MSCs were transfected with MR75 nanoparticles (determined after physicochemical assessment), delivering 0.5 µg, 1 µg, and 2 µg of pLuc. These doses were chosen based on previous work carried out in the literature [58].

siRNA: siGLO red transfection indicator (Dharmacon, Horizon, London, UK) containing a Dy-547 tag was used to assess the association of LDH–siRNA complexes with MSCs.

miRNA: fluorescent Dy-547 tagged scrambled miRNA mimics, or hairpin inhibitors (Dharmacon, Horizon, London, UK) were used to assess the association of LDH–miRNA complexes with MSCs.

Flow cytometry (Attune NxT, Thermo Fisher, Germany) was used to assess cellular uptake of fluorescently tagged LDH particles. Before analysis, cells were washed with a 100 µg/mL solution of heparin to remove any extracellular complexes. Cells were washed in PBS, trypsinized and fixed in 10% formalin before being suspended in PBS. A single-cell suspension was then analyzed for Dy-547 (Cy3 analog) fluorescence, and the percentage of cells associated with a red fluorescent signal was expressed as percentage internalization efficiency.

#### 2.3.2. LDH–Nucleic Acid Cytocompatibility

Cell proliferation following transfection was assessed by measuring cell number using the Quant-iT PicoGreen dsDNA kit (BioSciences, Dublin, Ireland) according to the manufacturer’s protocol. Cell number was compared to untreated control cells 7 days after transfection with LDH nanoparticles. Briefly, 100 μL of 1X PicoGreen reagent solution was added to samples lysed in 0.2 M carbonate and 1% Triton-X100 (BioSciences, Dublin, Ireland) cell lysate buffer. Fluorescence was read at 538 nm using a VarioskanFlash plate reader (Thermo Scientific, Germany) and SkanIt RE for VarioSkan software. The final DNA (pg/mL) concentration was calculated from the standard curve generated using standards formulated according to the manufacturer’s instructions.

Cell metabolic activity was measured using an alamarBlue^®^ assay (BioSciences, Dublin, Ireland) at days 1, 3, and 7 post-transfection. The alamarBlue^®^ assay was carried out as per the manufacturer’s instructions, where cells were incubated with 10% alamarBlue^®^ for two hours before fluorescent measurements were taken at an excitation wavelength of 545 nm and an emission wavelength of 590 nm. Results were normalized using media alone incubated with alamarBlue^®^.

#### 2.3.3. Assessment of LDH–Nucleic Acid Nanoparticle Efficacy in 2D

pDNA-LDH nanoparticle transfection efficiency was assessed by measuring levels of *Gaussia* luciferase in cell supernatant at 3 days post-transfection, using the Pierce™ *Gaussia* luciferase Flash Assay Kit (Fisher Scientific Ireland, Dublin, Ireland) as per the manufacturer’s instructions. Luminescence was read on a Tecan Infinite^®^200 Pro plate reader. Cells transfected with Lipofectamine^®^3000-pLuc as per the manufacturer’s instructions were used as the positive control. Untransfected cells were used as the negative control.

LDH–siRNA functionality and transfection efficiency was carried out using quantitative real-time PCR to assess target mRNA levels following the delivery of siRNA. RNA extraction of cells was carried out using the RNeasy mini kit (Qiagen, Manchester, UK). RNA quantity and quality were assessed using a NanoDrop. Reverse transcription of the RNA was carried out using a QuantiTect reverse transcription kit as per the manufacturer’s instructions (Qiagen, Manchester, UK). DNA was added to PCR plates at a concentration of 5 ng/µl along with required primers and SYBRGreen. The following predesigned and bioinformatically validated QuantiTect primer assays (Qiagen, Manchester, UK) were used; GAPDH (QT01082004), 18S (QT02589300). RNA fold change was assessed using the ∆∆Ct method and normalized to 18S.

The expression of miRNA was assessed using the miRNeasy mini kit (Qiagen, Manchester, UK) as per the manufacturer’s protocol. MiRNA was reverse transcribed into cDNA using the Taqman advanced cDNA synthesis kit (BioSciences, Dublin, Ireland) and miRNA advanced primer assays (rno-miR-16-5p targeting the mature miRNA sequence UAGCAGCACGUAAAUAUUGGCG and rno-miR-21-5p targeting the mature miRNA sequence UAGCUUAUCAGACUGAUGUUGA; BioSciences, Dublin, Ireland). Quantitative real-time PCR for the targeted rno-microRNA-16–5p and rno-microRNA-21–5p as a stably expressed endogenous control was performed using TaqMan Advanced miRNA assays and Taqman master mix (BioSciences, Dublin, Ireland).

### 2.4. LDH–Nanoparticle Activated Collagen-Nanohydroxyapatite Scaffold Systems

Coll-nHA scaffolds were fabricated with a 1:1 ratio collagen:nanohydroxyapatite as described previously [4,6,59,60]. Coll-nHA slurry was freeze-dried, physically crosslinked and sterilized through a dehydrothermal (DHT) treatment, at 105 °C for 24 h at a pressure of approximately 0.5 bar, cut into cylinders of 8 mm-diameter and chemically crosslinked in 1-(3-dimethylaminopropyl)-3-ethylcarbodiimide hydrochloride (EDAC, Sigma-Aldrich, Arklow, Ireland) and N-hydroxysuccinimide. Scaffolds were soak-loaded on each side, in 15-min incubation steps, with 25 μL LDH–nucleic acid nanoparticles at a final concentration of 50 nM of siRNA/miRNA following the procedure described in Section 2.2.

### 2.5. Confocal Imaging

Confocal imaging was performed to visualize uptake of nucleic acids into MSCs cultured on gene-activated collagen-nanohydroxyapatite scaffolds. Dy547-tagged miRNAs were visualized within the cytoplasm of cells on collagen-nHA scaffolds. The nucleus was stained with Hoechst stain, and the cytoplasm was stained using a green HCS CellMask™ according to the manufacturer’s protocol (BioSciences, Dublin, Ireland). Images were acquired on a Carl Zeiss LSM 710, equipped with a W Plan-Apochromat 20× (NA 1.0) and W N-Achroplan 10× (NA 0.3) objective. Images were prepared in FIJI.

### 2.6. Scanning Electron Microscopy

The microstructure of coll–nHA scaffolds was visualized by scanning electron microscopy (SEM). Non-loaded 8 mm-diameter scaffolds were fixed, prepared by ethanol and acetone dehydration and critical point drying. Scaffolds were then sputter-coated with palladium/gold using a Cressington 108 Coater. Imaging was carried out using a Carl Zeiss Ultra SEM operated at 5 kV in secondary electron mode.

### 2.7. Statistical Analysis

Data analysis was performed using GraphPad Prism (v8). Results were presented as the mean ± standard deviation and subjected to a one-way analysis of variance (ANOVA) and Tukey’s post hoc comparison. Cell metabolic activity changes at days 1, 3, and 7 post-transfection were analyzed using a two-way ANOVA. All experiments were performed in triplicate. ≤ 0.05 was considered a significant difference where * *p* < 0.05, ** *p* < 0.01, *** *p* < 0.001, **** *p* < 0.0001.

## 3. Results

### 3.1. Efficiency of LDH–pDNA Nanoparticle Complexation

**Figure 1:**
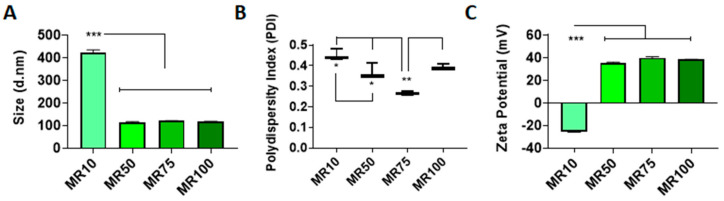
Electrostatic interaction of layered double hydroxide (LDH) with nucleic acids (pDNA) resulted in the formation of LDH–pDNA nanoparticles of varying physicochemical properties depending on the formulation. (**A**) LDH–pDNA nanoparticles ≥ MR50 were less than 200 nm in size. (**B**) MR75 nanoparticles were shown to have a significantly lower polydispersity index (PDI) compared with other formulations. (**C**) LDH–pDNA nanoparticles ≥ MR50 demonstrated a positively charged zeta potential (mV). Graphs represent mean ± std dev.; all measurements were carried out in triplicate. * = *p* < 0.05, ** = *p* < 0.01, *** = *p* < 0.001.

**Figure A2:**
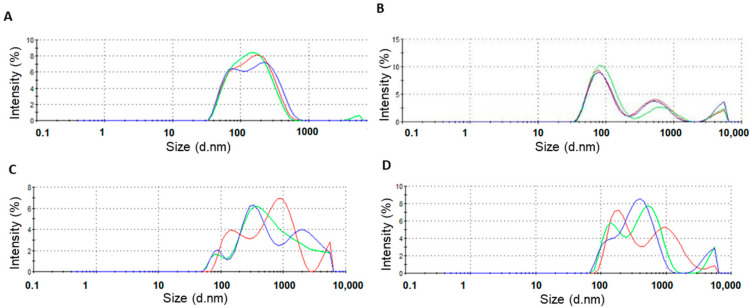
Representative size distribution curves of (**A**) LDH–pDNA nanoparticles of MR75, (**B**) siRNA-LDH nanoparticles of MR10, (**C**) miRNA mimic-LDH nanoparticles of MR10 and (**D**) miRNA inhibitor–LDH nanoparticles of MR10. Each line represents one repeat of a triplicate.

Size, polydispersity and zeta potential measurements of nanoparticles were analyzed to determine if nanoparticles had appropriate properties for cellular uptake. LDH–pDNA nanoparticles were formulated at MR10, 50, 75 and 100, based on previous reports [42]. Nanoparticles ≥ MR50 had a hydrodynamic diameter of <200 nm, a polydispersity index in the range of 0.26 to 0.44 and a positive zeta potential in the range of 35.0–39.9 mV (Figure 1A–C). Nanoparticles formulated at MR10 had a negative zeta potential, suggesting that anionic pDNA was present in excess of cationic LDH (Figure 1C) and had not successfully formed cationic nanoparticles. MR75 nanoparticles had a significantly lower polydispersity index (PDI) than the other groups (** *p* < 0.01), indicating a more homogenous sample with a lower propensity to aggregate. Therefore, the MR75 formulation was taken forward for testing in the cell transfection studies. Size distribution curves of the MR75 formulation are shown in Appendix A Figure A2A. The encapsulation efficiency (%) of LDH–pDNA nanoparticles at MR50, 75, and 100 was shown to be zero (data not shown). The assay used was based on the detection of uncomplexed pDNA, which we hypothesized may be detecting DNA bound to the outer layer of the LDH, rather than in the interlayer, as described in other sources in the literature in the discussion section. As the other characterization methods used in this study indicated the successful formation of particles, it was decided to continue and test the capability of LDH nanoparticles for pDNA cell transfection at MR75.

### 3.2. Efficiency of LDH–siRNA Complexation and Encapsulation

**Figure 2:**
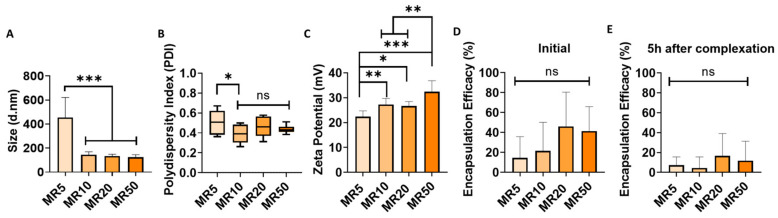
Electrostatic interaction of LDH with small, linear nucleic acids (siRNA) resulted in the formation of LDH–siRNA complexes of varying physicochemical characteristics dependent on the formulation. Physicochemical characterization of LDH–siRNA complexes with varying mass ratios (LDH:siRNA) demonstrated (**A**) a significant decrease in complex size at higher ratios (MR10, 20 and 50) compared to MR5. (**B**) All complexes formed demonstrated a positive zeta potential. (**C**) Higher mass ratios demonstrated a significantly lower polydispersity index (PDI) compared to MR5. (**D**) Encapsulation efficacy was demonstrated for all mass ratios, with a trend in increased encapsulation observed with increasing mass ratio. (**E**) All formulations demonstrated a decrease in encapsulation efficacy following a 5 h incubation period at 37 °C. Graphs represent mean ± std dev.; all measurements were carried out in triplicate. * = *p* < 0.05, ** = *p* < 0.01, *** = *p* < 0.001.

Successful complexation of cationic LDH with anionic siRNA resulted in the formation of LDH–siRNA complexes of varying size depending on the mass ratio of LDH–siRNA. A range of mass ratios was assessed for complexation efficiency with siRNA based on previous reports of LDH complexation with siRNA, albeit using a different composition of LDH to the one used in this study [61]. Following the complexation of LDH with siRNA, all formulations examined resulted in complexes of < 600 nm in diameter (Figure 2A). Increasing the mass ratio (more LDH compared to siRNA) demonstrated a significant reduction in the hydrodynamic diameter of the LDH–siRNA complexes formed for MR10, MR20 and MR50, all exhibiting a mean hydrodynamic diameter of < 200 nm (Figure 2A), which is conducive to transfection [62]. Size distribution curves of the MR10 formulation are shown in Appendix A Figure A2B.

All formulations of LDH–siRNA complexes exhibited a positive zeta potential ranging between 22 and 33 mV (Figure 2B). Increasing the ratio of LDH:siRNA from MR5 to MR50 resulted in a significant increase in the zeta potential of complexes formed. Additionally, the polydispersity index (Figure 2C) of the complexes formulated was shown to decrease with an increase in the ratio of LDH–siRNA with MR10, MR20 and MR50, all exhibiting a lower degree of polydispersity compared to MR5. No significant difference in polydispersity was observed between MR10, MR20 and MR50.

LDH–siRNA complexes demonstrated varying degrees of encapsulation efficacy (Figure 2D), with a trend in increasing encapsulation efficacy observed with an increase in the mass ratio. MR20 and MR50 demonstrated the highest levels of initial encapsulation efficacy of siRNA. The lowest mass ratio of MR5 demonstrated the lowest initial encapsulation efficacy. The stability of LDH–siRNA complexes was determined based on encapsulation efficacy (%) measured after 5 h. Following initial complexation, complexes were stored at 37 °C for 5 h, representative of the conditions to be used during transfection. All complex formulations demonstrated a reduction in the encapsulation efficacy of siRNA cargo 5 h after complexation (Figure 2E).

The resulting physicochemical data indicated that all the mass ratios assessed in this study were capable of successfully forming LDH–siRNA complexes. While there was no significant difference in encapsulation efficacy between each formulation, a clear trend was observed with an increase in encapsulation efficacy associated with the higher mass ratios. Taking into account the smaller size, polydispersity and higher zeta potential, all of which should improve the cellular uptake efficiency of the LDH–siRNA complexes, as well as the acceptable encapsulation efficacy, mass ratios at MR10 and MR20 were taken forward for further assessment of their in vitro transfection efficiency and cytocompatibility potential. MR50 nanoparticles were not selected as the higher amount of LDH would likely show adverse effects on cell viability as previously documented in the literature [42] in addition to choosing a more cost-effective approach.

### 3.3. Efficiency of LDH–miRNA Complexation and Encapsulation

**Figure 3:**
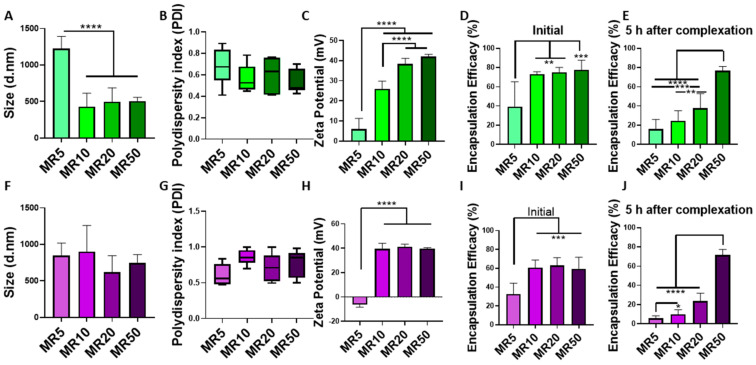
Physicochemical characterization of LDH–miRNA and LDH–hairpin inhibitor complexes. Characterization of miRNA mimics with LDH at MR5, 10, 20 and 50 showed (**A**) a significant decrease in size for MR10, 20 and 50 compared to MR5, (**B**) no difference in polydispersity index and, (**C**) a significant increase in zeta potentials for MR10, 20 and 50 compared to MR5. (**D**) Encapsulation efficacy showed successful encapsulation for MR10, 20 and 50 and (**E**) decrease of encapsulated miRNA mimics over 5 h at 37 °C for all MRs except MR50. Characterization of miRNA inhibitors with LDH at MR5, 10, 20 and 50 showed (**F**) no difference in size, (**G**) polydispersity index, and (**H**) significantly higher zeta potentials for MR10, 20 and 50 compared to MR5. (**I**) Successful encapsulation was demonstrated for MR10, 20 and 50 and (**E**) a decrease of encapsulated miRNA inhibitors over 5 h at 37 °C for all MRs except MR50. Graphs represent mean ± std dev.; all measurements were carried out in triplicate. * = *p* < 0.05, ** = *p* < 0.01, *** = *p* < 0.001, **** = *p* < 0.0001.

LDH was found to successfully complex with both miRNA mimics and inhibitors for MRs higher than 10 and resulted in nanoparticles with a size ranging between 429 and 903 nm in diameter. Size distribution curves of the MR10 formulations for miRNA mimics and miRNA inhibitors are shown in Appendix A Figure A2C,D. There was a significant decrease in size for LDH–mimic nanoparticles with increasing MR (Figure 3A), but no difference in size between LDH–inhibitor nanoparticles at different MRs (Figure 3F). The polydispersity index was not significantly different for the various formulations for either miRNA mimic or inhibitor nanoparticles (Figure 3B,G). The nanoparticles had a positive zeta potential for the three higher MRs (MR10, 20 and 50) compared to the MR5 nanoparticles that were negatively charged for hairpin inhibitors (Figure 3H) and positively, but significantly lower for MR5 than all other tested MRs for miRNA mimics (Figure 3C). LDH was found to encapsulate miRNA mimics at MR10 (73%), 20 (75%) and 50 (76%) (Figure 3D) and hairpin inhibitors at MR10 (61%), 20 (63%) and 50 (60%) (Figure 3I), whereas MR5 ratios led to a significantly lower encapsulation efficiency of only 39% for mimics and 33% for hairpin inhibitors. Stability was measured after complexes were stored at 37 °C for 5 h, which reflects the incubation time that was used for transfections. The percentage of encapsulated mimics decreased to 16% for MR5, 25% for MR10, 38% for MR20, and increased to 77% for MR50 after 5 h (Figure 3E). For miRNA inhibitors, encapsulation after 5 h decreased to 6% (MR5), 10% (MR10) and 24% (MR20) and increased to 71% for MR50. Results showed MR50 nanoparticles were more stable over 5 h as compared to the lower MRs tested. Based on these results, MR10 and MR20 LDH–nanoparticles were chosen for further assessment.

### 3.4. Uptake and Cytocompatibility of LDH–pDNA Nanoparticles in Monolayer Transfection

**Figure 4:**
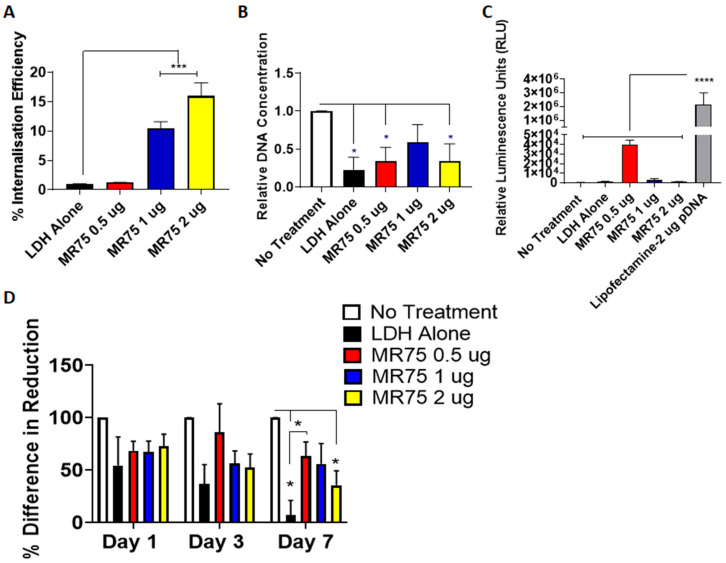
Cytocompatibility, uptake and transfection efficiency of LDH–pDNA complexes in 2D (**A**) Quantitative assessment of internalization, determined by flow cytometry, showed a significant increase in the internalization of LDH–pDNA complexes at 1 µg and 2 µg doses. (**B**) Cell proliferation, quantified by fold change in DNA content, was reduced in LDH treated groups, demonstrating the cytotoxic effect of LDH–pDNA complexes. (**C**) Transfection efficiency of LDH–pLuc complexes, assessed by luciferase assay, showed negligible levels of transfection compared to Lipofectamine^®^3000. (**D**) Cell metabolic activity was significantly reduced in LDH alone, LDH-0.5 µg, and LDH-2 µg pDNA treated cells at day 7 post-transfection. Graphs represent mean ± std dev.; all measurements were carried out in triplicate. * = *p* < 0.05, *** = *p* < 0.001, **** = *p* < 0.0001.

The efficacy of LDH as a pDNA delivery vector was assessed for nanoparticle internalization and transfection efficiency. Nanoparticle uptake in MSCs was determined using Cy3-tagged LDH–pDNA nanoparticles and flow cytometry, measured at day 3 post-transfection. Nanoparticles delivering 1 µg and 2 µg of pDNA demonstrated significantly greater internalization efficiency than those delivering 0.5 µg, as shown in Figure 4A. However, as pDNA transfection occurs in the nucleus, uptake does not necessarily translate to transfection and subsequent expression of the desired gene. The effect of LDH–pDNA nanoparticles on cell proliferation, quantified by fold change in DNA content was next determined (Figure 4B). DNA content was significantly lower in cells treated with LDH alone, MR75 nanoparticles delivering 0.5 µg, and 2 µg pDNA compared with untreated cells. To verify functionality, MSCs were transfected with pLuc to quantify levels of transfection (Figure 4C). All LDH–pDNA nanoparticles showed minimal transfection levels compared to the commercial gold standard Lipofectamine 3000^®^, indicating that LDH nanoparticles were not successfully transfecting cells with pDNA. Collectively, these results indicated that LDH-pDNA nanoparticles were internalized, but did not successfully transfect cells with pLuc.

Cell metabolic activity was then measured on days 1, 3 and 7 post-transfection (Figure 4D) and showed significantly reduced metabolic activity in LDH alone and LDH–2 µg pDNA transfected groups, compared with no treatment controls at day 7. Additionally, LDH-0.5 µg pDNA nanoparticles demonstrated significantly higher cell metabolic activity compared to LDH alone at day 7.

Based on the detrimental effect of LDH–pDNA nanoparticles on DNA content and cell metabolic activity, combined with low transfection efficiency, these nanoparticles were not taken forward for future work in scaffold-based transfections.

### 3.5. Uptake and Cytocompatibility of LDH–SiRNA Nanoparticles in Monolayer Transfection

**Figure 5:**
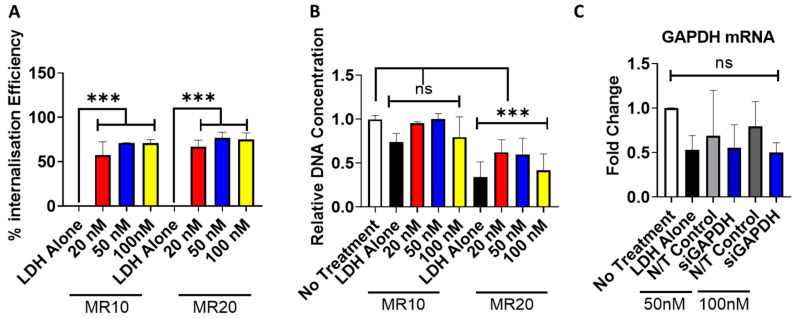
Cellular uptake and cytocompatibility analysis of LDH–siRNA complexes. (**A**) Quantitative assessment of cellular uptake, determined by flow cytometry, indicated successful uptake of LDH–siRNA complexes at both formulations and all concentrations (20 nM, 50 nM and 100 nM). (**B**) PicoGreen dsDNA assay analysis indicated cytocompatibility of LDH–siRNA complexes was dependent on formulation with higher amounts of LDH (MR20) exhibiting a significant reduction in DNA content compared to MR10 (7 days post-transfection). (**C**) Assessment of transfection efficiency and LDH–siRNA functionality by qPCR for the relative fold change of GAPDH compared to 18S in mesenchymal stromal cells (MSCs) (MR10) delivering 50 nM and 100 nM of siRNA. Graphs represent mean ± std dev.; all measurements were carried out in triplicate. *** = *p* < 0.001.

Having shown that LDH can encapsulate siRNA, MR10 and MR20 formulations were evaluated in terms of cytocompatibility, cellular uptake and functionality in 2D monolayer transfection. Quantitative analysis of the cellular uptake and internalization efficiency of fluorescently tagged siRNA, carried out using flow cytometry, confirmed the successful delivery and cellular uptake of LDH–siRNA complexes at both MR10 and MR20 (Figure 5A). Both LDH–siRNA complexes (MR10 and MR20) demonstrated successful cellular uptake at 20 nM.

Cytocompatibility analysis of LDH–siRNA complexes was assessed to determine cytotoxic effects of varying mass ratios of LDH–siRNA complexes as well as any siRNA dose-dependent cytotoxic effects. Cytocompatibility was found to be dependent on the mass ratio. Complexes formulated at MR10 exhibited no cytotoxic effects as determined by DNA content compared to the no treatment control group (Figure 5B). Complexes formulated at the higher mass ratio (MR20) resulted in a significant decrease in DNA content for each dose of siRNA delivered compared to the no treatment control.

The functionality of LDH–siRNA complexes was investigated through the delivery of siRNA targeting GAPDH (Figure 5C). LDH complexes containing siRNA-GAPDH resulted in a decrease in GAPDH mRNA at 24 h post-transfection. RNA knockdown was observed with the delivery of both 50 nM and 100 nM, GAPDH siRNA doses resulting in a reduction in fold expression compared to the no treatment control group and the delivery of a non-targeting (N/T) siRNA at 50 nM and 100 nM. Although demonstrating efficient cellular uptake and a favorable cytocompatibility profile, delivery of siGAPDH using MR10 did not result in a significant knockdown of GAPDH mRNA in MSCs. Similarly, in a system using MG_2_AL-Cl LDH nanoparticles complexed with siRNA/double-stranded DNA, Wu et al. detected MR20 as an optimal formulation of LDH–siRNA nanoparticles for cellular uptake but found favorable functionality for MR5 formulations [61].** The delivery of LDH alone (at equivalent amounts required to deliver 100 nM siRNA) demonstrated a reduction in GAPDH mRNA, which may be a result of the toxicity effects observed with the delivery of LDH alone (Figure 5B).

### 3.6. Uptake and Cytocompatibility of LDH–MiRNA Mimic and LDH–MiRNA Inhibitor Nanoparticles in Monolayer Transfection

**Figure 6:**
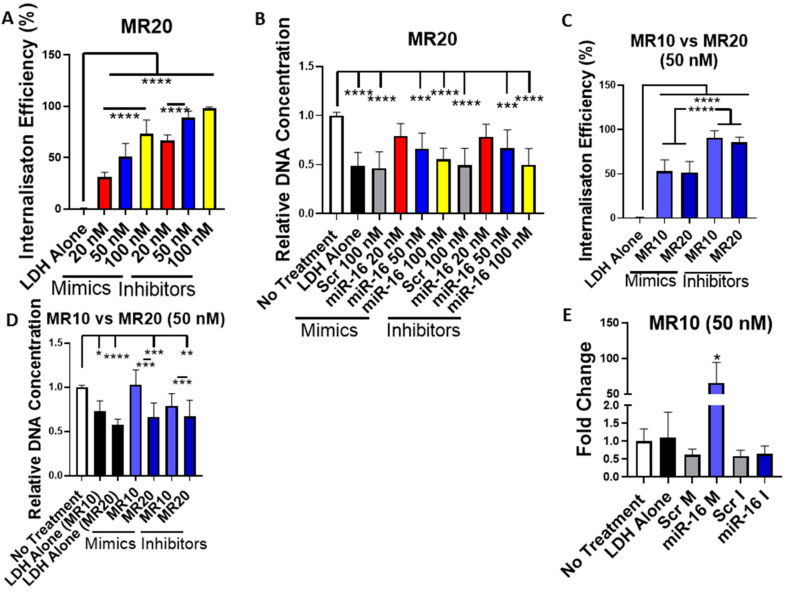
Cytotoxicity, uptake and transfection efficiency of LDH–miRNA mimic and LDH–hairpin inhibitor complexes in 2D (**A**) flow cytometry analysis for comparison of internalization efficiency after transfection with LDH at MR20 and different doses (20 nM, 50 nM, 100 nM) for mimics and hairpin inhibitors showed increased internalization for higher doses of both, and lower internalization efficiency for LDH–mimic nanoparticles compared to LDH–inhibitor nanoparticles. (**B**) PicoGreen dsDNA assay showed that both LDH–mimic and LDH–inhibitor MR20 nanoparticles had adverse effects on DNA content that increased with higher miRNA doses. There was no significant difference between the effects of LDH–mimic versus LDH–inhibitor nanoparticles. (**C**) flow cytometry analysis for comparison of internalization efficiency after transfection with LDH at MR10 and MR20 at a 50 nM dose for mimics and hairpin inhibitors showed no significant difference based on MR used while LDH–mimic treatment resulted in a lower internalization efficiency compared to LDH–inhibitor transfection. (**D**) PicoGreen dsDNA assay showing that MR10 LDH–mimic and LDH–inhibitor nanoparticles had no significant effect on DNA content assessed at 7 days post-transfection as compared to untreated controls while MR20 LDH–mimics and inhibitor nanoparticles significantly decreased cell viability. (**E**) qPCR for miRNA-16 showed that LDH–mimic delivery induced a significant increase in miRNA-16 expression, whereas hairpin inhibitor delivery had no significant effect on miRNA-16 expression. Data were analyzed by ∆∆Ct method and normalized to miRNA-21. Graphs represent mean ± std dev., all measurements were carried out in triplicate. Scr: scrambled, M: mimic, I: inhibitor. * = *p* < 0.05, ** = *p* < 0.01, *** = *p* < 0.001, **** = *p* < 0.0001.

Having shown that LDH can efficiently encapsulate miRNA mimics and hairpin inhibitors, MR10 and MR20 formulations were selected for further assessment in 2D monolayer culture regarding cellular uptake, functionality and effect on cell viability. The uptake of fluorescently tagged mimics and hairpin inhibitors was quantified by flow cytometry (Figure 6A). A dose-dependent increase in internalization efficiency was found after transfection with MR20 nanoparticles. However, mimic and hairpin inhibitor delivery using the MR20 formulation had an adverse effect on cell viability resulting in a significant decrease in DNA content when assessed 7 days after transfection (Figure 6B). This effect was present for nanoparticles delivering miRNA-16 targeting nucleic acids as well as for the non-targeting scrambled controls. As a result, the MR10 formulation was assessed to determine cellular uptake and cytotoxic effects. Interestingly, internalization efficiency for the MR10 formulation was equivalent to that measured for the MR20 formulation for both mimics and hairpin inhibitors (Figure 6C). Furthermore, transfection with MR10 nanoparticles had no adverse effect on cell viability compared to non-treated cells, indicating no inhibitory effect on the MSCs (Figure 6D). Owing to the high delivery efficacy and no impact on cell viability, MR10 was chosen as the optimal MR dose for future experiments. The functionality of the delivered mimics was confirmed by qPCR showing a significant increase in miRNA-16 expression 3 days post-transfection (Figure 6E). However, no significant decrease was detected in miRNA-16 expression after hairpin inhibitor delivery at the same time point, indicating that the hairpin inhibitor did not bind significant amounts of the endogenously expressed miRNA-16. Taken together, these results demonstrate that LDH can successfully deliver miRNA mimics into the cytoplasm of MSCs in monolayer culture without any adverse effects on cell viability.

### 3.7. SiRNA Delivery from Gene-Activated Collagen-Nanohydroxyapatite Scaffolds

**Figure 7:**
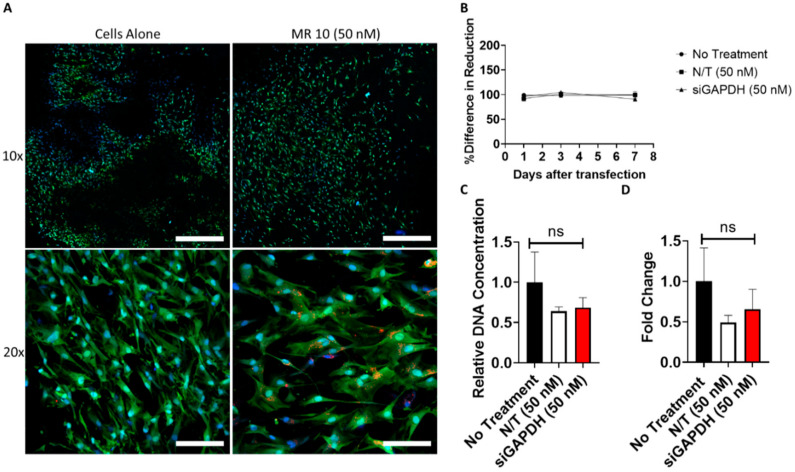
LDH–siRNA activated coll–nHA scaffolds demonstrate successful cellular uptake and functionality of siRNA with favorable cytocompatibility. (**A**) Confocal imaging of MSCs cultured on coll–nHA scaffolds with and without LDH–siGLO complexes (red)demonstrated successful cellular uptake of complexes exhibiting peri-nuclear localization of complexes. MSCs were stained with an HCS cell mask (green) for the cytoplasm and a Hoechst stain for the nucleus (blue). Scale bar = 10× = 600 µM, 20× = 100 µm. MSCs cultured on LDH–siRNA activated scaffolds exhibited no difference in (**B**) metabolic activity and (**C**) viability when compared to cells cultured on coll–nHA scaffolds without complexes. (**D**) Assessment of functionality of siRNA by qPCR for GAPDH RNA knockdown following transfection (Day 7) in MSCs seeded on coll–nHA scaffolds. RNA fold change was assessed using the ∆∆Ct method and normalized to 18S. Graphs represent mean ± std dev.; all measurements were carried out in triplicate.

**Figure A3:**
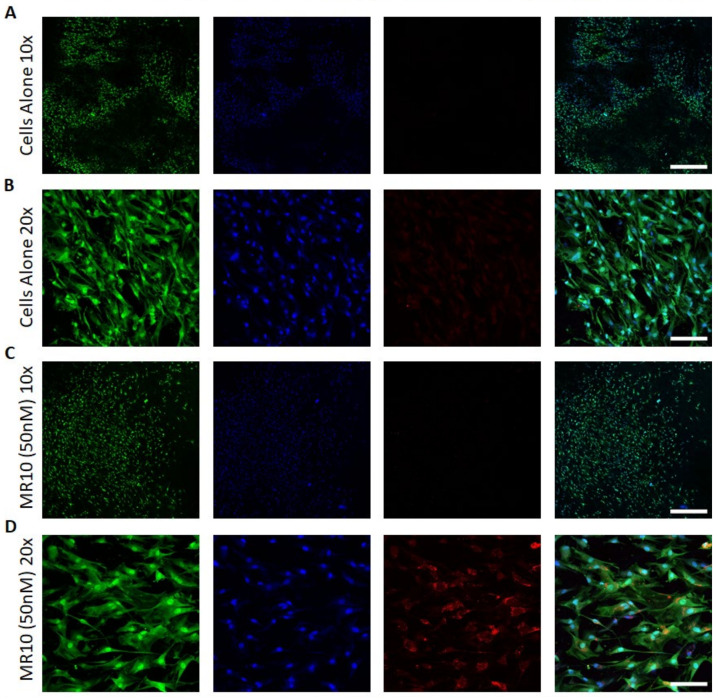
Separate channels of a confocal image of rat MSCs on a coll–nHA scaffold shown in Figure 7. MSCs were stained with an HCS cell mask (green) for the cytoplasm and a Hoechst stain for the nucleus (blue). Confocal images of scaffolds containing cells without treatment at (**A**) 10 × and (**B**) 20 × as well as MR10 LDH–siRNA (50nM) with a red-transfection indicator at (**C**) 10 × and (**D**) 20 ×. Scale bar = 10 × = 600 µM, 20 × = 100 µM.

Following the successful delivery of LDH–siRNA complexes demonstrating a favorable cytocompatibility profile (MR10), LDH–siRNA complexes were incorporated into a coll–nHA scaffold for the development of a gene-activated scaffold (Figure 7). Confocal imaging demonstrated successful incorporation of LDH–siRNA complexes (MR10–50nM) within the coll–nHA architecture (Figure 7A and Appendix A Figure A3) and highlighted the colocalization of fluorescently tagged LDH–siRNA complexes within the cell body, demonstrating peri-nuclear localization. The culturing of MSCs on siRNA-activated scaffolds had no negative effect on cell metabolic activity (Figure 7B) or DNA content (Figure 7C). The functionality of internalized LDH–siRNA complexes containing siRNA targeting GAPDH mRNA demonstrated a trend towards successful knockdown 7 days after seeding MSCs on LDH–siRNA-activated scaffolds, but this effect was not significant (Figure 7D). However, delivery of a non-targeting siRNA also demonstrated a reduction in GAPDH mRNA levels when assessed by qPCR.

### 3.8. MicroRNA Delivery from Gene-Activated Collagen-Nanohydroxyapatite Scaffolds

**Figure 8:**
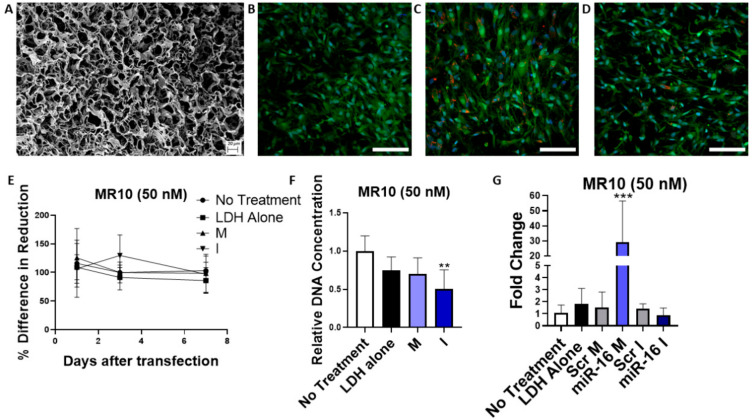
Assessment of MSC uptake, cytotoxicity and transfection on LDH–mimic and LDH–hairpin inhibitor loaded coll–nHA scaffolds. (**A**) scanning electron microscopy image of a coll–nHA scaffold. Scale bar = 20 μM. (**B**) Confocal image of MSCs on a coll–nHA scaffold. MSCs were stained with an HCS cell mask (green) for the cytoplasm and a Hoechst stain for the nucleus (blue). (**C**) Dy547-labeled scrambled mimics or (**D**) hairpin inhibitors (red) were observed within the MSC cytoplasm on the LDH–nanoparticle loaded scaffolds. Scale bar = 100 μM (**E**) No adverse metabolic activity effects were demonstrated over 7 days. (**F**) DNA quantification indicated that cell number was maintained across all groups except the LDH–inhibitor loaded scaffolds; LDH–mimic nanoparticles showed no treatment-associated cytotoxic effects 7 days after seeding. ** = *p* < 0.01 (**G**) LDH–mimic activated scaffolds induced a significant overexpression of miRNA-16 7 days post-transfection. PCR data were analyzed by the ∆∆Ct method and normalized to miRNA-21. Graphs represent mean ± std dev.; all measurements were carried out in triplicate. Scr: scrambled, M: mimic, I: inhibitor., *** = *p* < 0.001.

**Figure A4:**
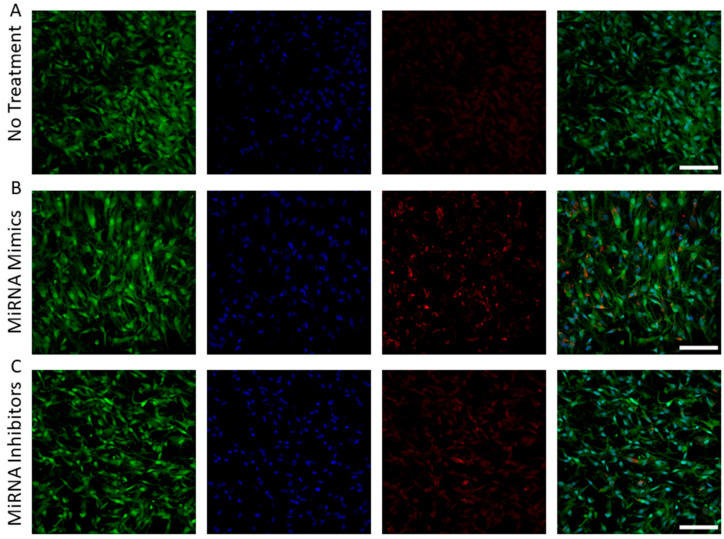
Separate channels of a confocal image of rat MSCs on a coll–nHA scaffold shown in Figure 8. MSCs were stained with an HCS cell mask (green) for the cytoplasm and a Hoechst stain for the nucleus (blue). (**A**) Cells without treatment transfected, (**B**) with a red-tagged miRNA-mimic or (**C**) inhibitor. Scale bar = 100 μM.

Having evaluated the efficacy of nucleic acid delivery by LDH nanoparticles in monolayer, the ability to deliver miRNA from gene activated coll–nHA scaffolds incorporating LDH–nanoparticles was assessed. Figure 8A shows the coll–nHA scaffolds imaged by SEM demonstrating an interconnected porous structure. Confocal images showed the MSCs grown for 7 days on a coll–nHA scaffolds (Figure 8B and Appendix A Figure A4A) and internalization of red LDH–Dy547-mimic (Figure 8C and Appendix A Figure A4B) and hairpin inhibitors (Figure 8D and Appendix A Figure A4C). Cell metabolic activity was not affected by culturing on miRNA-activated collagen scaffolds (Figure 8E). DNA content was significantly reduced for hairpin inhibitor-loaded scaffolds but was unaffected when MSCs were cultured on miRNA mimic-activated scaffolds indicating no adverse impact of LDH–mimic nanoparticles on cell viability of MSCs on coll–nHA scaffolds (Figure 8F). The functionality of internalized LDH–mimic nanoparticles was confirmed by a significant upregulation of miRNA-16 levels 7 days after seeding MSCs on the scaffolds (Figure 8G), while LDH–hairpin inhibitor activated scaffolds did not result in a significant downregulation of miRNA-16 level. This indicates that the hairpin inhibitor did not bind significant amounts of miRNA-16 within the cytoplasm of the MSCs and correlates with the qPCR results detected after miRNA transfection in 2D. These results indicate that while LDH–inhibitor activated coll–nHA scaffolds failed to induce significant functionality of miRNA inhibitors, LDH nanoparticles can successfully be utilized as delivery platforms for encapsulated miRNA-mimics from coll–nHA scaffolds leading to significant overexpression of the delivered miRNA-16 while maintaining cell viability.

## 4. Discussion

LDHs are two-dimensional biocompatible materials composed of cationic layers and anionic interlayers that have previously been used as nonviral vectors for gene and drug delivery [63]. This study characterizes LDH as a nonviral delivery vector for different nucleic acids, including pDNA, siRNA and miRNA mimics and hairpin inhibitors. The composition of LDH (MgAl-NO_3_) was capable of encapsulating nucleic acids.

While LDH–pDNA nanoparticles were internalized by MSCs at higher doses, they demonstrated poor transfection efficiency and high levels of cytotoxicity. LDH was capable of complexing with pDNA to form positively charged nanoparticles at MR50 and higher. However, MR75 pDNA nanoparticles were shown to be significantly less polydisperse than other formulations tested, suggesting that these nanoparticles may be more uniform in size and, therefore, more suitable for cell uptake and subsequent transfection. However, the encapsulation efficiency was zero for all LDH–pDNA nanoparticles and did not increase in correlation with increasing MR. As the LDH–pDNA binding mechanism is unknown, we hypothesized that the encapsulation assay in this instance is potentially detecting pDNA bound to the LDH surface layers, and thus may not be fully encapsulated. This hypothesis correlates with other findings in the literature [64,65], which describe the adsorption of DNA to the surface of MgAl and LDH–lactate nanosheets, respectively. Additionally, the successful complexation of LDH and pDNA has shown to be heavily reliant on the LDH formulation used, as well as the fabrication process. Sanderson et al. have previously shown that hydrotalcite LDHs synthesized at higher temperatures tend to form tighter associations with pDNA, which is thought to be due to the closer matching of charge densities between larger LDH particles and pDNA [66]. Collectively, these reports concur with our findings that the same LDH formulation, which successfully complexes with siRNA and miRNA, may not complex with larger, double-stranded pDNA. While LDH–pDNA complexes at higher doses appeared to be taken up by MSCs, transfection levels measured by Gaussia luciferase activity were minimal compared to the commercial gold standard Lipofectamine, as expected given that LDH was not shown to successfully encapsulate pDNA. While there are some reports in the literature of successful pDNA transfection in HEK 293T cells [52], myoblasts by utilizing Zn/Al LDH [67], NIH-3T3 and HeLa cells using Li-Al LDH [53], and mouse motor neuron cells using [(Mg3Al(OH)8](CO3)0.5] [54], our findings showed that LDH nanoparticles in the MgAl-NO3 formulation were unsuitable for pDNA transfection in MSCs. Additionally, there are no reports in the literature of successful LDH–pDNA transfection in MSCs thus far.

However, this study demonstrated successful delivery of small, linear nucleic acids (siRNA, miRNA mimics and hairpin inhibitors) to MSCs with complexes exhibiting a favorable cytocompatibility profile. LDH–miRNA mimic nanoparticles showed functionality in monolayer culture, indicating successful release of the nucleic acids into the cytoplasm without exhibiting adverse effects on cell viability. Overall, the results indicate that LDH is a suitable nonviral vector for miRNA mimics and demonstrates the promising potential for the delivery of siRNA following efficient encapsulation and cellular uptake. Furthermore, LDH–miRNA activated coll–nHA scaffolds were shown to upregulate miRNA expression in MSCs and therefore, the developed biomaterial scaffolds provide a potential delivery platform for therapeutic applications in regenerative medicine.

Delivery of siRNA and miRNA (MR10 and MR20) demonstrated successful cellular uptake of LDH in a dose-dependent manner with the successful delivery of nucleic acid cargoes at 20 nM, 50 nM and 100 nM. These doses have previously been shown to be suitable to induce functional delivery of siRNA and miRNA [4,68] and correspond to an amount of 0.1 to 1 µg of nucleic acids, therefore being in the same range of the pDNA assessed for transfection. Further to this, the delivery of these smaller, linear nucleic acids, namely miRNA mimics, demonstrated functionality, indicating successful release of the biomolecules from the LDH once internalization has occurred. Previous studies suggest that the dissolution of the LDH transfection agent within the endosome following internalization plays a role in aiding endosomal escape, releasing the cargo into the cytoplasm of the cell [34]. It has also been reported that dissolution of the LDH transfection carrier results in the production of “cytofriendly” ions and no evidence of carrier accumulation inside the cell, which is often the case for inorganic or polymeric nonviral vectors [34,69]. However, this “cytofriendly effect” may be dependent on several factors, including the amount of LDH used during complexation as well as the different LDH materials available for testing (e.g., chloride or nitrate based LDH–precursors). Even though the internalization of LDH–hairpin inhibitors was greater than that of LDH–mimic inhibitors, delivery of hairpin inhibitors did not induce a significant downregulation of the endogenous miRNA. This could be due to challenges in measuring miRNA expression that could disguise small expression changes. Measuring miRNA expression by qPCR requires an additional amplification step that can induce nonlinearity in the measurement [70]. Discrepancies between optimal formulations for nucleic acid nanoparticle internalization and functionality could furthermore be caused by effects on nucleic acid release into the cytoplasm. Although demonstrating efficient cellular uptake and a favorable cytocompatibility profile, delivery of siGAPDH using MR10 did not result in a significant knockdown of GAPDH mRNA in MSCs. Similarly, in a system using MG2AL-Cl LDH nanoparticles complexed with siRNA/double-stranded DNA, Wu et al. detected MR20 as an optimal formulation for cellular uptake but found favorable functionality for MR5 formulations [61]. Increased stability of nanoparticles achieved by a higher MR can have negative effects on intracellular release, as has been shown for other vector systems such as chitosan-pDNA nanoparticles [71].

Successful formulation of LDH–complexes at the lower MR10 demonstrated a significant reduction in the cytotoxic effects observed in cells treated compared to a no treatment control. These results suggest that LDH–nanoparticles can exhibit cytotoxicity when LDH is concentrated above a certain threshold, but, at lower amounts, LDH is not cytotoxic. This is supported by the literature, and although some groups report little cytotoxicity associated with LDH nanoparticles following delivery to mammalian cells, others report varying degrees of the threshold amount of LDH [72,73]. Concerning the broad application of LDH as a nonviral vector for gene therapy, the size of the nucleic acid being delivered (pDNA, siRNA, miRNA) plays a key role in the success of delivery. The results outlined in this study indicate that LDH is a suitable delivery vector for small nucleic acids such as siRNA and miRNA mimics [74,75,76]. LDH has previously been described for the delivery of miRNA mimics to macrophages [77], and it has been reported that layered gadolinium hydroxychloride can successfully deliver anti-miRNA oligonucleotides to inhibit miRNA-10b in breast cancer cells [78] but has not, to our knowledge, been optimized for the delivery of miRNA mimics and inhibitors to MSCs. Therefore, this is the first study showing the potential application of LDH as a nonviral delivery vector to induce miRNA mediated RNAi in MSCs. The safe and transient delivery of nucleic acids to the cytoplasm without undesirable translocation to the nucleus is a challenging approach, as nonviral vectors tend to have a low transfection efficacy as well as cytotoxic effects, whereas viral gene delivery induces genomic integration and leads to long-lasting changes in gene expression. Therefore, identifying a nonviral vector for small nucleic acids, such as the LDH described in this study, capable of inducing RNAi with high efficacy and low toxicity provides a particularly useful, safe, and transient tool for gene therapy applications.

In addition to the cellular barriers that face the successful delivery of nucleic acid cargoes to cells, there are several “extracellular” barriers that hinder successful delivery of the desired therapeutic to the target cells, including susceptibility to serum degradation, rapid clearance from the site of action, short timeframe, and requirement for repeated administration of the therapeutic. Biomaterial-based scaffold delivery systems offer the potential to overcome these limitations while also allowing for spatiotemporal control over the delivery of the therapeutic. While miRNA-activated coll–nHA scaffolds have been reported for bone repair applications [5,7,79], this study demonstrates the first biomaterial-based delivery of nucleic acids to MSCs using an LDH–activated scaffold delivery system. Several scaffold-based systems incorporating the use of various formulations of LDH have been reported in the literature. For example, LDH–chitosan scaffolds loaded with Pifithrin α (PFTα), a selective p53 inhibitor, have previously been utilized for bone regeneration applications in vitro and in vivo [80]. Ag-loaded MgSrFe–LDH/chitosan scaffolds have been shown to increase osteogenic marker expression as well as ALP activity of human bone marrow-MSCs through the controlled release of strontium [81]. This study has characterized LDH as a potent nonviral vector for nontoxic and cost-effective delivery of small nucleic acids, such as siRNA and miRNA mimics, to MSCs in monolayer cell culture as well as from biocompatible coll–nHA scaffolds, indicating its potential for use for gene therapy applications.

## 5. Conclusions

This study demonstrates the successful use of a layered double hydroxide as an efficient delivery vector for short, linear, and small nucleic acids (siRNA, miRNA mimics and hairpin inhibitors) to MSCs without cytotoxic effects. The incorporation of LDH–nucleic acid complexes within a collagen-based delivery platform, previously optimized within our group for bone repair [2,4,27], demonstrates successful localized transfection of MSCs bearing great potential for tissue regeneration applications. We thereby demonstrate the successful use of LDH for the safe and effective delivery of various nucleic acids with potential applications in many tissue engineering and regenerative medicine approaches.

## Figures and Tables

**Figure A1:**
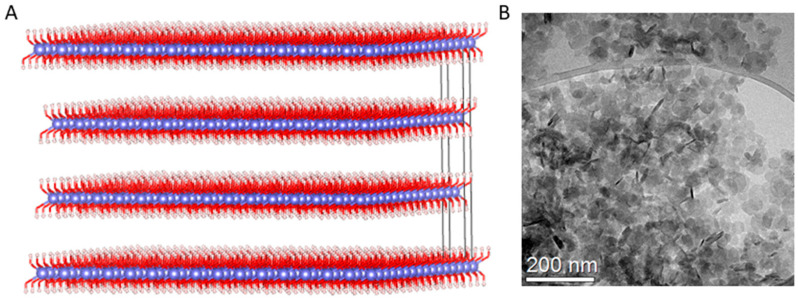
(**A**) Schematic of the cationic layers of the LDH crystal structure. Divalent/trivalent metals, oxygen and hydrogen, are presented in blue, red and white, respectively. (**B**) The morphological features of the Mg-Al LDH material were characterized using transmission electron microscopy. Samples were prepared by placing 3 μL of LDH nanoparticle solution on Lacey carbon TEM grids (Ted Pella, USA) and baked at 60° for 24 h. TEM of the samples was performed on an FEI Titan operating at 300 kV (Thermo Fisher Scientific, USA).

## References

[1] Naldini L. (2015). Gene therapy returns to centre stage. Nature.

[2] Curtin C.M., Tierney E.G., McSorley K., Cryan S.A., Duffy G.P., O’Brien F.J. (2015). Combinatorial gene therapy accelerates bone regeneration: Non-viral dual delivery of VEGF and BMP2 in a collagen-nanohydroxyapatite scaffold. Adv. Healthc. Mater..

[3] Kelly D.C., Raftery R.M., Curtin C.M., O’Driscoll C.M., O’Brien F.J. (2019). Scaffold-Based Delivery of Nucleic Acid Therapeutics for Enhanced Bone and Cartilage Repair. J. Orthop. Res. Off. Publ. Orthop. Res. Soc..

[4] Mencía Castaño I., Curtin C.M., Shaw G., Murphy J.M., Duffy G.P., O’Brien F.J. (2015). A novel collagen-nanohydroxyapatite microRNA-activated scaffold for tissue engineering applications capable of efficient delivery of both miR-mimics and antagomiRs to human mesenchymal stem cells. J. Control. Release Off. J. Control. Release Soc..

[5] Castaño I.M., Raftery R.M., Chen G., Cavanagh B., Quinn B., Duffy G.P., O’Brien F.J., Curtin C.M. (2020). Rapid bone repair with the recruitment of CD206(+)M2-like macrophages using non-viral scaffold-mediated miR-133a inhibition of host cells. Acta Biomater..

[6] Curtin C.M., Cunniffe G.M., Lyons F.G., Bessho K., Dickson G.R., Duffy G.P., O’Brien F.J. (2012). Innovative collagen nano-hydroxyapatite scaffolds offer a highly efficient non-viral gene delivery platform for stem cell-mediated bone formation. Adv. Mater..

[7] Mencía Castaño I., Curtin C.M., Duffy G.P., O’Brien F.J. (2016). Next generation bone tissue engineering: Non-viral miR-133a inhibition using collagen-nanohydroxyapatite scaffolds rapidly enhances osteogenesis. Sci. Rep..

[8] Raftery R.M., Gonzalez Vazquez A.G., Chen G., O’Brien F.J. (2020). Activation of the SOX-5, SOX-6, and SOX-9 Trio of Transcription Factors Using a Gene-Activated Scaffold Stimulates Mesenchymal Stromal Cell Chondrogenesis and Inhibits Endochondral Ossification. Adv. Healthc. Mater..

[9] Yan L.P., Castaño I.M., Sridharan R., Kelly D., Lemoine M., Cavanagh B.L., Dunne N.J., McCarthy H.O., O’Brien F.J. (2020). Collagen/GAG scaffolds activated by RALA-siMMP-9 complexes with potential for improved diabetic foot ulcer healing. Mater. Sci. Eng. C Mater. Biol. Appl..

[10] Laiva A.L., O’Brien F.J., Keogh M.B. (2020). SDF-1α gene-activated collagen scaffold drives functional differentiation of human Schwann cells for wound healing applications. Biotechnol. Bioeng..

[11] Liu H., Yang Z., Xun Z., Gao Z., Sun Y., Yu J., Yang T., Zhao X., Cai C., Ding P. (2019). Nuclear delivery of plasmid DNA determines the efficiency of gene expression. Cell Biol. Int..

[12] Bartel D.P. (2009). MicroRNAs: Target recognition and regulatory functions. Cell.

[13] Leng Q., Chen L., Lv Y. (2020). RNA-based scaffolds for bone regeneration: Application and mechanisms of mRNA, miRNA and siRNA. Theranostics.

[14] Sadakierska-Chudy A. (2020). MicroRNAs: Diverse Mechanisms of Action and Their Potential Applications as Cancer Epi-Therapeutics. Biomolecules.

[15] Arabian M., Mirzadeh Azad F., Maleki M., Malakootian M. (2020). Insights into role of microRNAs in cardiac development, cardiac diseases, and developing novel therapies. Iran. J. Basic Med. Sci..

[16] Bajan S., Hutvagner G. (2020). RNA-Based Therapeutics: From Antisense Oligonucleotides to miRNAs. Cells.

[17] Fire A., Xu S., Montgomery M.K., Kostas S.A., Driver S.E., Mello C.C. (1998). Potent and specific genetic interference by double-stranded RNA in Caenorhabditis elegans. Nature.

[18] Urits I., Swanson D., Swett M.C., Patel A., Berardino K., Amgalan A., Berger A.A., Kassem H., Kaye A., Viswanath O. (2020). A Review of Patisiran (ONPATTRO^®^) for the Treatment of Polyneuropathy in People with Hereditary Transthyretin Amyloidosis. Neurol. Ther..

[19] Helal N.A., Osami A., Helmy A., McDonald T., Shaaban L.A., Nounou M.I. (2017). Non-viral gene delivery systems: Hurdles for bench-to-bedside transformation. Pharmazie.

[20] Benskey M.J., Sandoval I.M., Miller K., Sellnow R.L., Gezer A., Kuhn N.C., Vashon R., Manfredsson F.P. (2019). Basic Concepts in Viral Vector-Mediated Gene Therapy. Methods Mol. Biol..

[21] Heyde M., Partridge K.A., Oreffo R.O.C., Howdle S.M., Shakesheff K.M., Garnett M.C. (2007). Gene therapy used for tissue engineering applications. J. Pharm. Pharmacol..

[22] Bono N., Ponti F., Mantovani D., Candiani G. (2020). Non-Viral in Vitro Gene Delivery: It is Now Time to Set the Bar!. Pharmaceutics.

[23] Guo X., Huang L. (2012). Recent advances in nonviral vectors for gene delivery. Acc. Chem. Res..

[24] Patil S., Gao Y.G., Lin X., Li Y., Dang K., Tian Y., Zhang W.J., Jiang S.F., Qadir A., Qian A.R. (2019). The Development of Functional Non-Viral Vectors for Gene Delivery. Int. J. Mol. Sci..

[25] Cullis P.R., Hope M.J. (2017). Lipid Nanoparticle Systems for Enabling Gene Therapies. Mol. Ther. J. Am. Soc. Gene Ther..

[26] Kulkarni J.A., Cullis P.R., van der Meel R. (2018). Lipid Nanoparticles Enabling Gene Therapies: From Concepts to Clinical Utility. Nucleic Acid Ther..

[27] Raftery R.M., Tierney E.G., Curtin C.M., Cryan S.A., O’Brien F.J. (2015). Development of a gene-activated scaffold platform for tissue engineering applications using chitosan-pDNA nanoparticles on collagen-based scaffolds. J. Control. Release Off. J. Control. Release Soc..

[28] Dixon J.E., Osman G., Morris G.E., Markides H., Rotherham M., Bayoussef Z., El Haj A.J., Denning C., Shakesheff K.M. (2016). Highly efficient delivery of functional cargoes by the synergistic effect of GAG binding motifs and cell-penetrating peptides. Proc. Natl. Acad. Sci. USA.

[29] Cryan S.A., Holohan A., Donohue R., Darcy R., O’Driscoll C.M. (2004). Cell transfection with polycationic cyclodextrin vectors. Eur. J. Pharm. Sci. Off. J. Eur. Fed. Pharm. Sci..

[30] Elsabahy M., Nazarali A., Foldvari M. (2011). Non-viral nucleic acid delivery: Key challenges and future directions. Curr. Drug Deliv..

[31] Bai H., Lester G.M.S., Petishnok L.C., Dean D.A. (2017). Cytoplasmic transport and nuclear import of plasmid DNA. Biosci. Rep..

[32] Hu H., Xiu K.M., Xu S.L., Yang W.T., Xu F.J. (2013). Functionalized layered double hydroxide nanoparticles conjugated with disulfide-linked polycation brushes for advanced gene delivery. Bioconjug. Chem..

[33] Hobbs C., Jaskaniec S., McCarthy E.K., Downing C., Opelt K., Güth K., Shmeliov A., Mourad M.C.D., Mandel K., Nicolosi V. (2018). Structural transformation of layered double hydroxides: An in situ TEM analysis. NPJ 2D Mater. Appl..

[34] Xu Z.P., Niebert M., Porazik K., Walker T.L., Cooper H.M., Middelberg A.P., Gray P.P., Bartlett P.F., Lu G.Q. (2008). Subcellular compartment targeting of layered double hydroxide nanoparticles. J. Control. Release Off. J. Control. Release Soc..

[35] Choi S.J., Oh J.M., Choy J.H. (2010). Biocompatible nanoparticles intercalated with anticancer drug for target delivery: Pharmacokinetic and biodistribution study. J. Nanosci. Nanotechnol..

[36] Kim J.Y., Choi S.J., Oh J.M., Park T., Choy J.H. (2007). Anticancer drug-inorganic nanohybrid and its cellular interaction. J. Nanosci. Nanotechnol..

[37] Choy J.H., Jung J.S., Oh J.M., Park M., Jeong J., Kang Y.K., Han O.J. (2004). Layered double hydroxide as an efficient drug reservoir for folate derivatives. Biomaterials.

[38] Oh J.M., Choi S.J., Kim S.T., Choy J.H. (2006). Cellular uptake mechanism of an inorganic nanovehicle and its drug conjugates: Enhanced efficacy due to clathrin-mediated endocytosis. Bioconjug. Chem..

[39] Kriven W., Kwak S.Y., Wallig M., Choy J.H. (2004). Bio-Resorbable Nanoceramics for Gene and Drug Delivery. MRS Bull. Mater. Res. Soc..

[40] Ladewig K., Niebert M., Xu Z.P., Gray P.P., Lu G.Q. (2010). Efficient siRNA delivery to mammalian cells using layered double hydroxide nanoparticles. Biomaterials.

[41] Desigaux L., Belkacem M.B., Richard P., Cellier J., Léone P., Cario L., Leroux F., Taviot-Guého C., Pitard B. (2006). Self-assembly and characterization of layered double hydroxide/DNA hybrids. Nano Lett..

[42] Xu Z.P., Walker T.L., Liu K.L., Cooper H.M., Lu G.Q., Bartlett P.F. (2007). Layered double hydroxide nanoparticles as cellular delivery vectors of supercoiled plasmid DNA. Int. J. Nanomed..

[43] Choi S.J., Oh J.M., Choy J.H. (2008). Safety Aspect of Inorganic Layered Nanoparticles: Size-Dependency In Vitro and In Vivo. J. Nanosci. Nanotechnol..

[44] Kim K.M., Lim S.K. (2014). Role of miRNAs in bone and their potential as therapeutic targets. Curr. Opin. Pharmacol..

[45] Cunniffe G.M., Dickson G.R., Partap S., Stanton K.T., O’Brien F.J. (2010). Development and characterisation of a collagen nano-hydroxyapatite composite scaffold for bone tissue engineering. J. Mater. Sci. Mater. Med..

[46] Fayyazbakhsh F., Solati-Hashjin M., Keshtkar A., Shokrgozar M.A., Dehghan M.M., Larijani B. (2017). Novel layered double hydroxides-hydroxyapatite/gelatin bone tissue engineering scaffolds: Fabrication, characterization, and in vivo study. Mater. Sci. Eng. C Mater. Biol. Appl..

[47] Fayyazbakhsh F., Solati-Hashjin M., Keshtkar A., Shokrgozar M.A., Dehghan M.M., Larijani B. (2017). Release behavior and signaling effect of vitamin D3 in layered double hydroxides-hydroxyapatite/gelatin bone tissue engineering scaffold: An in vitro evaluation. Colloids Surf. B Biointerfaces.

[48] Li L., Zhang R., Gu W., Xu Z.P. (2018). Mannose-conjugated layered double hydroxide nanocomposite for targeted siRNA delivery to enhance cancer therapy. Nanomed. Nanotechnol. Biol. Med..

[49] Park D.H., Cho J., Kwon O.J., Yun C.O., Choy J.H. (2016). Biodegradable Inorganic Nanovector: Passive versus Active Tumor Targeting in siRNA Transportation. Angew. Chem..

[50] Li L., Gu W., Chen J., Chen W., Xu Z.P. (2014). Co-delivery of siRNAs and anti-cancer drugs using layered double hydroxide nanoparticles. Biomaterials.

[51] Zheng W., Yin T., Chen Q., Qin X., Huang X., Zhao S., Xu T., Chen L., Liu J. (2016). Co-delivery of Se nanoparticles and pooled SiRNAs for overcoming drug resistance mediated by P-glycoprotein and class III β-tubulin in drug-resistant breast cancers. Acta Biomater..

[52] Wang J., Bao W., Umar A., Wang Q., O’Hare D., Wan Y. (2016). Delaminated Layered Double Hydroxide Nanosheets as an Efficient Vector for DNA Delivery. J. Biomed. Nanotechnol..

[53] Senapati S., Sarkar T., Das P., Maiti P. (2019). Layered Double Hydroxide Nanoparticles for Efficient Gene Delivery for Cancer Treatment. Bioconjug. Chem..

[54] Li S., Li J., Wang C.J., Wang Q., Cader M.Z., Lu J., Evans D.G., Duan X., O’Hare D. (2013). Cellular uptake and gene delivery using layered double hydroxide nanoparticles. J. Mater. Chem. B.

[55] Wong Y., Markham K., Xu Z.P., Chen M., Max Lu G.Q., Bartlett P.F., Cooper H.M. (2010). Efficient delivery of siRNA to cortical neurons using layered double hydroxide nanoparticles. Biomaterials.

[56] Yang H.Y., van Ee R.J., Timmer K., Craenmehr E.G.M., Huang J.H., Öner F.C., Dhert W.J.A., Kragten A.H.M., Willems N., Grinwis G.C.M. (2015). A novel injectable thermoresponsive and cytocompatible gel of poly(N-isopropylacrylamide) with layered double hydroxides facilitates siRNA delivery into chondrocytes in 3D culture. Acta Biomater..

[57] González-Vázquez A., Planell J.A., Engel E. (2014). Extracellular calcium and CaSR drive osteoinduction in mesenchymal stromal cells. Acta Biomater..

[58] Tierney E.G., Duffy G.P., Hibbitts A.J., Cryan S.A., O’Brien F.J. (2012). The development of non-viral gene-activated matrices for bone regeneration using polyethyleneimine (PEI) and collagen-based scaffolds. J. Control. Release Off. J. Control. Release Soc..

[59] Cunniffe G.M., Curtin C.M., Thompson E.M., Dickson G.R., O’Brien F.J. (2016). Content-Dependent Osteogenic Response of Nanohydroxyapatite: An in Vitro and in Vivo Assessment within Collagen-Based Scaffolds. ACS Appl. Mater. Interfaces.

[60] Curtin C.M., Castaño I.M., O’Brien F.J. (2018). Scaffold-Based microRNA Therapies in Regenerative Medicine and Cancer. Adv. Healthc. Mater..

[61] Wu Y., Gu W., Chen C., Do S.T., Xu Z.P. (2018). Optimization of Formulations Consisting of Layered Double Hydroxide Nanoparticles and Small Interfering RNA for Efficient Knockdown of the Target Gene. ACS Omega.

[62] Faraji A.H., Wipf P. (2009). Nanoparticles in cellular drug delivery. Bioorg. Med. Chem..

[63] Yan L., Gonca S., Zhu G., Zhang W., Chen X. (2019). Layered double hydroxide nanostructures and nanocomposites for biomedical applications. J. Mater. Chem. B.

[64] Andrea K.A., Wang L., Carrier A.J., Campbell M., Buhariwalla M., Mutch M., MacQuarrie S.L., Bennett C., Mkandawire M., Oakes K. (2017). Adsorption of Oligo-DNA on Magnesium Aluminum-Layered Double-Hydroxide Nanoparticle Surfaces: Mechanistic Implication in Gene Delivery. Langmuir ACS J. Surf. Colloids.

[65] Bao W., Wang J., Wang Q., O’Hare D., Wan Y. (2016). Layered Double Hydroxide Nanotransporter for Molecule Delivery to Intact Plant Cells. Sci. Rep..

[66] Sanderson B.A., Sowersby D.S., Crosby S., Goss M., Lewis L.K., Beall G.W. (2013). Charge density and particle size effects on oligonucleotide and plasmid DNA binding to nanosized hydrotalcite. Biointerphases.

[67] Yazdani P., Mansouri E., Eyvazi S., Yousefi V., Kahroba H., Hejazi M.S., Mesbahi A., Tarhriz V., Abolghasemi M.M. (2019). Layered double hydroxide nanoparticles as an appealing nanoparticle in gene/plasmid and drug delivery system in C_2_C1_2_ myoblast cells. Artif. Cells Nanomed. Biotechnol..

[68] Fitzgerald K.A., Guo J., Raftery R.M., Castaño I.M., Curtin C.M., Gooding M., Darcy R., FJ O.B., CM O.D. (2016). Nanoparticle-mediated siRNA delivery assessed in a 3D co-culture model simulating prostate cancer bone metastasis. Int. J. Pharm..

[69] Ladewig K., Xu Z.P., Lu G.Q. (2009). Layered double hydroxide nanoparticles in gene and drug delivery. Expert Opin. Drug Deliv..

[70] Chugh P., Dittmer D.P. (2012). Potential pitfalls in microRNA profiling. Wiley Interdiscip. Rev. RNA.

[71] Huang M., Fong C.W., Khor E., Lim L.Y. (2005). Transfection efficiency of chitosan vectors: Effect of polymer molecular weight and degree of deacetylation. J. Control. Release Off. J. Control. Release Soc..

[72] Ladewig K., Niebert M., Xu Z.P., Gray P.P., Lu G.Q. (2010). Controlled preparation of layered double hydroxide nanoparticles and their application as gene delivery vehicles. Appl. Clay Sci..

[73] Choi S.J., Oh J.M., Choy J.H. (2009). Toxicological effects of inorganic nanoparticles on human lung cancer A549 cells. J. Inorg. Biochem..

[74] Chen M., Cooper H.M., Zhou J.Z., Bartlett P.F., Xu Z.P. (2013). Reduction in the size of layered double hydroxide nanoparticles enhances the efficiency of siRNA delivery. J. Colloid Interface Sci..

[75] Acharya R., Chakraborty M., Chakraborty J. (2019). Prospective treatment of Parkinson’s disease by a siRNA-LDH nanoconjugate. MedChemComm.

[76] Wong Y., Cooper H.M., Zhang K., Chen M., Bartlett P., Xu Z.P. (2012). Efficiency of layered double hydroxide nanoparticle-mediated delivery of siRNA is determined by nucleotide sequence. J. Colloid Interface Sci..

[77] Yang L., Sun J., Liu Q., Zhu R., Yang Q., Hua J., Zheng L., Li K., Wang S., Li A. (2019). Synergetic Functional Nanocomposites Enhance Immunotherapy in Solid Tumors by Remodeling the Immunoenvironment. Adv. Sci..

[78] Yoo S.S., Razzak R., Bédard E., Guo L., Shaw A.R., Moore R.B., Roa W.H. (2014). Layered gadolinium-based nanoparticle as a novel delivery platform for microRNA therapeutics. Nanotechnology.

[79] Mencía Castaño I., Curtin C.M., Duffy G.P., O’Brien F.J. (2019). Harnessing an Inhibitory Role of miR-16 in Osteogenesis by Human Mesenchymal Stem Cells for Advanced Scaffold-Based Bone Tissue Engineering. Tissue Eng. Part A.

[80] Chen Y.X., Zhu R., Ke Q.F., Gao Y.S., Zhang C.Q., Guo Y.P. (2017). MgAl layered double hydroxide/chitosan porous scaffolds loaded with PFTalpha to promote bone regeneration. Nanoscale.

[81] Cao D., Xu Z., Chen Y., Ke Q., Zhang C., Guo Y. (2018). Ag-loaded MgSrFe-layered double hydroxide/chitosan composite scaffold with enhanced osteogenic and antibacterial property for bone engineering tissue. J. Biomed. Mater. Res. Part B Appl. Biomater..

